# Viral infections and related fatal adverse events associated with complement inhibitors for PNH: a real-world pharmacovigilance analysis in FAERS

**DOI:** 10.3389/fphar.2025.1639685

**Published:** 2025-08-11

**Authors:** Jinman Zhong, Chang Chen, Yunman Xu, Yueping He, Jiewen Tan, Dan Xiong

**Affiliations:** Department of Hematology, The Eighth Affiliated Hospital, Southern Medical University (The First People’s Hospital of Shunde, Foshan), Foshan, China

**Keywords:** complement inhibitor, PNH, viral infection adverse events, fatal infectionrelated adverse events, real-world pharmacovigilance analysis

## Abstract

**Background:**

Complement inhibitors are increasingly utilized across various clinical indications, including the treatment of paroxysmal nocturnal hemoglobinuria (PNH). A thorough understanding of their adverse events (AEs) profiles, particularly regarding infections, is essential to ensure safe and effective treatment strategies.

**Objective:**

To characterize the real-world AEs profile of complement inhibitors in PNH, with a focus on viral infections characteristics and distinct fatality risk, of while exploring potential implications for viral prophylaxis and identifying risk factors associated with fatal infection-related adverse events.

**Methods:**

Complement inhibitor-associated AE cases reported in FAERS between 2004 and 2024 were included. Pharmacovigilance analyses (including Reporting Odds Ratio [ROR] and multiple other metrics) were employed to detect signals for adverse events, including viral infection. Time-to-onset analysis and logistic regression were used to assess temporal patterns and identify factors associated with viral infections and fatal outcomes.

**Results:**

Among 58,613 AE reports, 11,957 (20.4%) were infection-related, and 8.91% were fatal. Infection-related AEs constituted 11,957 cases, predominantly linked to C5 inhibitors. Pharmacovigilance analysis revealed significant disproportionality signals for viral infections (e.g., influenza, herpes zoster, gastroenteritis viral, viral infection). C5 inhibitors had higher cases numbers, but C3 inhibitors demonstrated a stronger signal intensity (ROR = 3.52, 95%CI: 2.54–4.89). Fatal viral AEs had a median time-to-event of 12 days, while non-fatal viral infections occurred later, with a median time-to-event of 187 days. Older age, higher body weight, and treatment initiation in later quarters were associated with reduced viral infection risk, while female was linked to slightly elevated risk. While viral infections were common concomitant AEs, the fatality rate specifically for viral infections was lower compared to other complement inhibitor-associated AEs. Advanced age (≥75 years), treatment initiation in the third quarter and C5 inhibitor use were identified as significant risk factors for fatal infectious outcomes, whereas female sex and higher body weight appeared protective.

**Conclusion:**

Complement inhibitors, particularly C3 agents, are associated with significant reporting of infectious AEs in FAERS, including specific viral infections like influenza and herpes. Early onset of viral AEs highlights the need for vigilance early in treatment. While advanced age and C5 use heighten mortality risk, the attenuated lethality of viral AEs suggests a distinct pathophysiological interplay warranting mechanistic study. The divergent risk profiles between C3 and C5 inhibitors underscore the need for personalized risk-benefit assessments in complement inhibition strategies.

## 1 Introduction

Complement is a critical component of innate and adaptive immunity, facilitating pathogen elimination through opsonization, chemotaxis, and direct lysis *via* the membrane attack complex (MAC) (Sarma and Ward, 2011). However, its dysregulation can drive pathological inflammation in diverse clinical contexts (Ostrycharz and Hukowska-Szematowicz, 2022). Therapeutic inhibition of the complement cascade has revolutionized the treatment landscape for a spectrum of complement-mediated disorders, including PNH, a rare and life-threatening disorder characterized by chronic intravascular hemolysis and an elevated risk of thrombosis (Garred et al., 2021). Agents targeting various components, such as C5 inhibitors (e.g., eculizumab, ravulizumab), proximal C3 inhibitors (e.g., pegcetacoplan), and Factor B inhibitors, have received FDA approval for the treatment of PNH, with further indications under investigation (Zhang et al., 2024; Hillmen et al., 2006; Lee et al., 2019). Recently, Factor D inhibitors, such as danicopan, have been authorized as adjunctive therapy for PNH (Gavriilaki et al., 2022), echoed with the expanding use of complement inhibitors in clinical settings.

Despite their therapeutic efficacy, complement inhibitors are associated with heightened infection risks due to the system’s integral role in microbial defense. The bacterial infection risks are well-established: eculizumab, for instance, carries a black-box warning for meningococcal infections, necessitating prophylactic vaccination (Zhang et al., 2024; McNamara et al., 2017). Severe infections with *Streptococcus* pneumoniae and *Haemophilus* influenzae have also been documented with both C3 and C5 inhibitors (Dingli et al., 2024; Jayaraman et al., 2024). In contrast, the virologic consequences of complement blockade remain underexplored. Complement contributes to antiviral immunity through opsonization, inflammation and modulation of adaptive responses (Stoermer and Morrison, 2011). Case reports of viral reactivations, such as progressive multifocal leukoencephalopathy (PML) due to JC virus in patients receiving complement inhibitors underscore the potential risks (Gómez-Cibeira et al., 2016). However, systematic evaluations of these risks across inhibitor classes and patient subgroups are lacking. Specifically, factors affecting the fatal adverse events related to infection in complement inhibitor use remain unclear. Addressing these knowledge gaps is critical to optimizing therapy and complication management among complement inhibitors users.

To address this gap, we leveraged the rich data of AR outcomes and events related to complement inhibitors used specifically for treating PNH, reported from 2004 to 2024 in the U.S. FDA Adverse Event Reporting System (FAERS) is a robust post-marketing surveillance database that aggregates spontaneous adverse event reports from clinicians, manufacturers, and patients (Zhou et al., 2023; Zhai et al., 2019). FAERS has proven effective in identifying rare or delayed adverse events, including those associated with immune checkpoint inhibitors and antipsychotics (Zhou et al., 2023; Shu et al., 2023). Our study takes a structured approach. First, we aim to provide a descriptive overview of the real-world AE profile associated with the established complement inhibitors (C3, C5, and Factor B inhibitors). Second, given known general infection risk in complement inhibitors use, we further assess the fatal risk profile of viral infection, aiming to inform potential viral prophylaxis strategies. Finally, to further inform clinical practice, we assess the risk factors associated with overall fatal infectious AEs (encompassing viral and other infections) in patients treated with these complement inhibitors. This will help identify patient populations at higher risk who may warrant closer monitoring or specific management strategies. Through comprehensive pharmacovigilance signal detection, temporal trend analysis, and multivariate risk modeling, this investigation seeks to enhance the understanding of complement inhibitor safety profiles and inform targeted risk mitigation strategies.

## 2 Materials and methods

### 2.1 Data source

We conducted a retrospective pharmacovigilance study to investigate viral infection and related fatal adverse events signals associated with complement system inhibitors using data from FAERS (Sakaeda et al., 2013). In this study, we included the reports submitted between the first quarter of 2004 and the fourth quarter of 2024 and focused on three classes of complement inhibitors: the C3 inhibitor (Pegcetacoplan), C5 inhibitors (Crovalimab, Eculizumab, Pozelimab, Ravulizumab), and Factor B inhibitor (Iptacopan). Factor D inhibitors were excluded from this analysis because, although recently introduced (Gavriilaki et al., 2022), they are not yet widely used in clinical settings, resulting in very limited case data in FAERS. To ensure comprehensive case retrieval, drug names, including synonyms and aliases obtained from the FDA Drugs database, were used to search FAERS. The inclusion was restricted to reports where the specified complement inhibitor was documented as the primary suspect (PS) agent. All reported adverse events were systematically coded using the Medical Dictionary for Regulatory Activities (MedDRA, version 27.1) (Brown et al., 1999). For initial signal detection of infectious events, Preferred Terms (PTs) under the MedDRA System Organ Class (SOC) “Infections and infestations” (primary SOC designation with flag set to “Yes”) were extracted. Subsequently, a broader assessment was conducted by evaluating safety signals across all reported PTs to provide a comprehensive analysis of viral infections and other potential associated adverse events.

### 2.2 Data processing and quality control

In line with the FDA guideline, we processed the raw data from FAERS using a standardized processing protocol to ensure data quality and integrity. Initial record extraction from the DEMO table included the PRIMARYID, CASEID, and FDA_DT fields. A primary deduplication step was performed by sorting the dataset sequentially by CASEID, FDA_DT, and PRIMARYID. For records sharing the same CASEID, the entry with the most recent FDA_DT was retained. If both CASEID and FDA_DT were identical, the record with the highest PRIMARYID was selected. A secondary, content-based deduplication process removed records exhibiting identical values across key fields (gender, age, country, event date, adverse event, drug, indication). Reports identified as invalid or incomplete were excluded. Furthermore, the dataset was cross-referenced with FDA quarterly deletion lists to remove withdrawn reports. To mitigate confounding by indication or pre-existing conditions, reports were excluded if the patient received systemic anti-infective therapy (antibiotics, antivirals, or antifungals) within 14 days prior to initiating complement inhibitor treatment. This criterion aimed to enhance the specificity of safety signal attribution by reducing potential misclassification of baseline infections as treatment-emergent adverse events.

### 2.3 Signal detection

Pharmacovigilance signal detection was conducted utilizing a comprehensive suite of eight established disproportionality analysis methods to ensure the robustness of findings (Beninger, 2018; Meyboom et al., 1997). The employed algorithms included: the Reporting Odds Ratio (ROR), the Proportional Reporting Ratio (PRR) (Evans et al., 2001), Fisher’s exact test, a frequentist Observed-to-Expected (O/E) ratio analysis, the Chi-square (χ^2^) test, two distinct implementations of the Bayesian Confidence Propagation Neural Network (BCPNN) – one utilizing normal approximation and the other employing Monte Carlo simulation (Bate et al., 1998) and the Empirical Bayes Geometric Mean (EBGM), often associated with the Multi-item Gamma Poisson Shrinker (MGPS) algorithm (Szarfman et al., 2002). Specific criteria defined signal presence for each method, generally following established practices. Notably, for both ROR and PRR, a signal was flagged if the lower bound of the 95% confidence interval (95% CI) exceeded 1, contingent upon a minimum frequency of three reports. The Chi-square (χ^2^) test required at least three reports, a PRR ≥2, and a χ^2^ statistic ≥4, while Fisher’s exact test indicated a signal with a p-value <0.05. For both BCPNN implementations, a signal was identified if the lower 95% CI bound for the Information Component (IC) exceeded 0 (IC025 > 0) (Bate et al., 1998). Similarly, the O/E ratio analysis flagged a signal if the lower 95% CI bound was greater than 0, and the EBGM analysis required the lower 95% CI bound (EBGM05) to exceed 2 (DuMouchel, 1999). All identified adverse event Preferred Terms within the dataset were systematically evaluated using these eight methodologies. To specifically classify a viral infection PT as potentially related to complement inhibitors, a stringent, multi-faceted criterion was applied, requiring both: (1) a positive ROR signal (lower 95% CI > 1, ≥3 reports) in the overall analysis and within analyses stratified by each inhibitor subclass (C3, C5, Factor B), and (2) at least one of the eight methods yielding a positive signal in the overall analysis and in at least two of the three subclass-specific analyses.

### 2.4 Statistical analysis

Baseline demographic and clinical characteristics of the study population, including age, sex, weight, complement inhibitor drug class, geographic region, and patient outcomes, were summarized using standard descriptive statistics, with medians and interquartile ranges (IQRs) for continuous variables and frequencies and percentages for categorical variables. The time-to-onset for complement inhibitor-associated viral infections was defined as the interval between therapy initiation and event onset. Given the absence of censoring as all identified cases experienced the event, a descriptive comparison of median time-to-onset and IQRs was performed across relevant subgroups. The associations of demographic and clinical characteristics with mortality (i.e., fatal vs. nonfatal) were examined using binary logistic regressions with estimates reported as Odds Ratios (ORs) and 95% CIs.

A subsequent stratified analysis evaluated the association between experiencing complement inhibitor-related viral infection PTs (defined *via* signal detection) and fatal outcomes within the cohort of all complement inhibitor recipients reporting adverse events, controlling for potential confounding factors. The primary exposure group comprised patients with these viral infection PTs, while the comparator group included patients with other PTs. Within strata defined by potential confounders (based on discretized continuous variables or original categorical levels), associations were assessed using 2 × 2 contingency tables, with Fisher’s exact test determining statistical significance and ORs with 95% CIs quantifying the association strength. This analysis employed a complete-case approach. Instances of sparse data, where any cell within a 2 × 2 table contained zero observations, were handled by applying the Haldane–Anscombe continuity correction (addition of 0.5 to all cells) for calculating the OR and its corresponding Woolf logit 95% CIs, while Fisher’s exact tests utilized the original, unadjusted cell counts.

Furthermore, potentially nonlinear relationships between continuous predictors (age, weight) and the probability of a fatal outcome were investigated using restricted cubic splines (RCS) (Arnes et al., 2023) within logistic regression models, adjusting for confounders (drug class, sex, and weight/age as appropriate). The degree of freedom of the RCS function (3–7 knots) was determined *via* Akaike Information Criterion (AIC) minimization (Arnes et al., 2023), and potential interactions between age/weight and sex were evaluated. Adjusted probability curves, based on median covariate values (age: 42 years; weight: 64.2 kg), were generated to visualize the functional form of these associations.

All statistical analyses were performed using R software (version 4.4.1), and statistical significance was set at p < 0.05 (two-tailed).

## 3 Results

### 3.1 Population characteristics of cases with complement inhibitor-related adverse events

**FIGURE 1:**
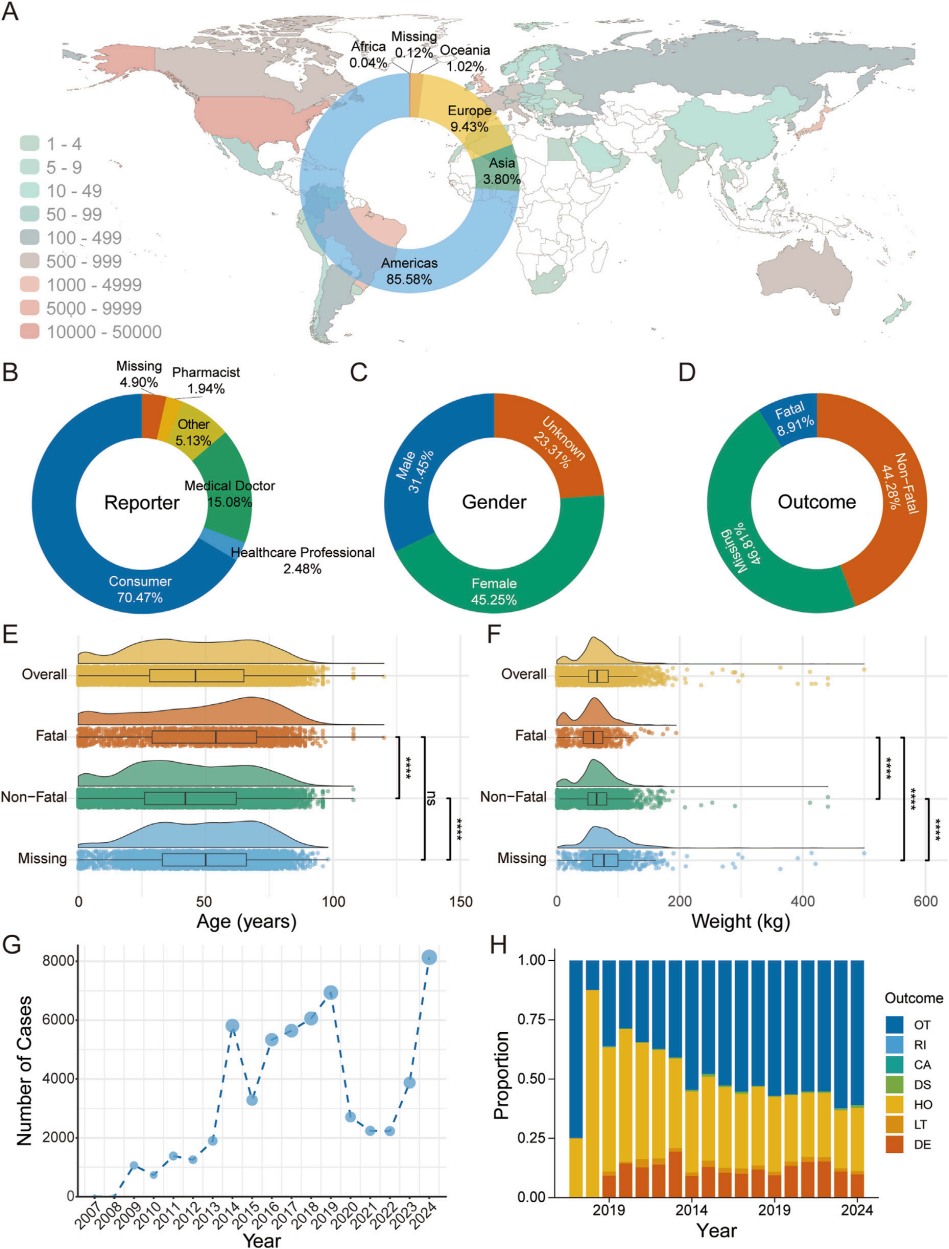
Descriptive Analysis of Adverse Events Associated with Complement Inhibitors Using FAERS Database. **(A)** Geographic distribution of adverse event reports across countries, with color indicating the number of reports; the donut chart illustrates the proportional distribution by continent. **(B)** Donut chart depicting the percentage distribution of adverse event reporters by professional identity. **(C)** Donut chart representing the gender distribution of individuals reporting adverse events. **(D)** Donut chart showing the proportion of adverse event outcomes. **(E)** Raincloud plot displaying the age distribution among the overall population, fatal group, non-fatal group, and missing outcome group. Group comparisons were performed using the Kruskal–Wallis test, with *P*-values adjusted by the Benjamini–Hochberg correction. Significance levels: ****P < 0.0001; ***P < 0.001; **P < 0.01; *P < 0.05; ns, *P* ≥ 0.05. **(F)** Raincloud plot illustrating the weight distribution among the overall population, fatal group, non-fatal group, and missing outcome group. Statistical differences were analyzed using the Kruskal–Wallis test, with *P*-values adjusted by the Benjamini–Hochberg correction. Significance levels: ****P < 0.0001; ***P < 0.001; **P < 0.01; *P < 0.05; ns, *P* ≥ 0.05. **(G)** Line graph presenting the annual number of adverse event cases associated with complement inhibitors. **(H)** Stacked bar chart demonstrating the temporal trends in the proportions of adverse event outcomes. Outcomes are categorized as follows: Death, LT: Life-Threatening, HO: Hospitalization - Initial or Prolonged, DS: Disability, CA: Congenital Anomaly, RI: Required Intervention to Prevent Permanent Impairment/Damage, OT: Other.

Among 58,613 complement inhibitor-associated AE reports in FAERS, 45.25% involved female patients, with most reports originating from consumers (70.47%) and physicians (15.08%) in the Americas (85.58%) (Figures 1A–C). Of the total reports, 8.91% (n = 5,222) documented fatal outcomes and 44.28% (n = 25,955) non-fatal outcomes (Figure 1D). Cases with fatal outcomes were older (mean age 48.97 vs 43.08 years) and paradoxically had lower body weight (mean 58.53 vs 65.50 kg) compared to non-fatal cases (Figures 1E,F, P < 0.05). Overall, the total number of AE reports showed an increasing trend over time, despite some year-to-year fluctuations and COVID-19 pandemic period from 2020–2022 (Figure 1G). Meanwhile, the proportion of non-serious AEs (classified as “OT”) also exhibited an overall upward trend, although with minor variations across years (Figure 1H).

### 3.2 Infectious adverse events among complement inhibitor users in FAERS

**FIGURE 2:**
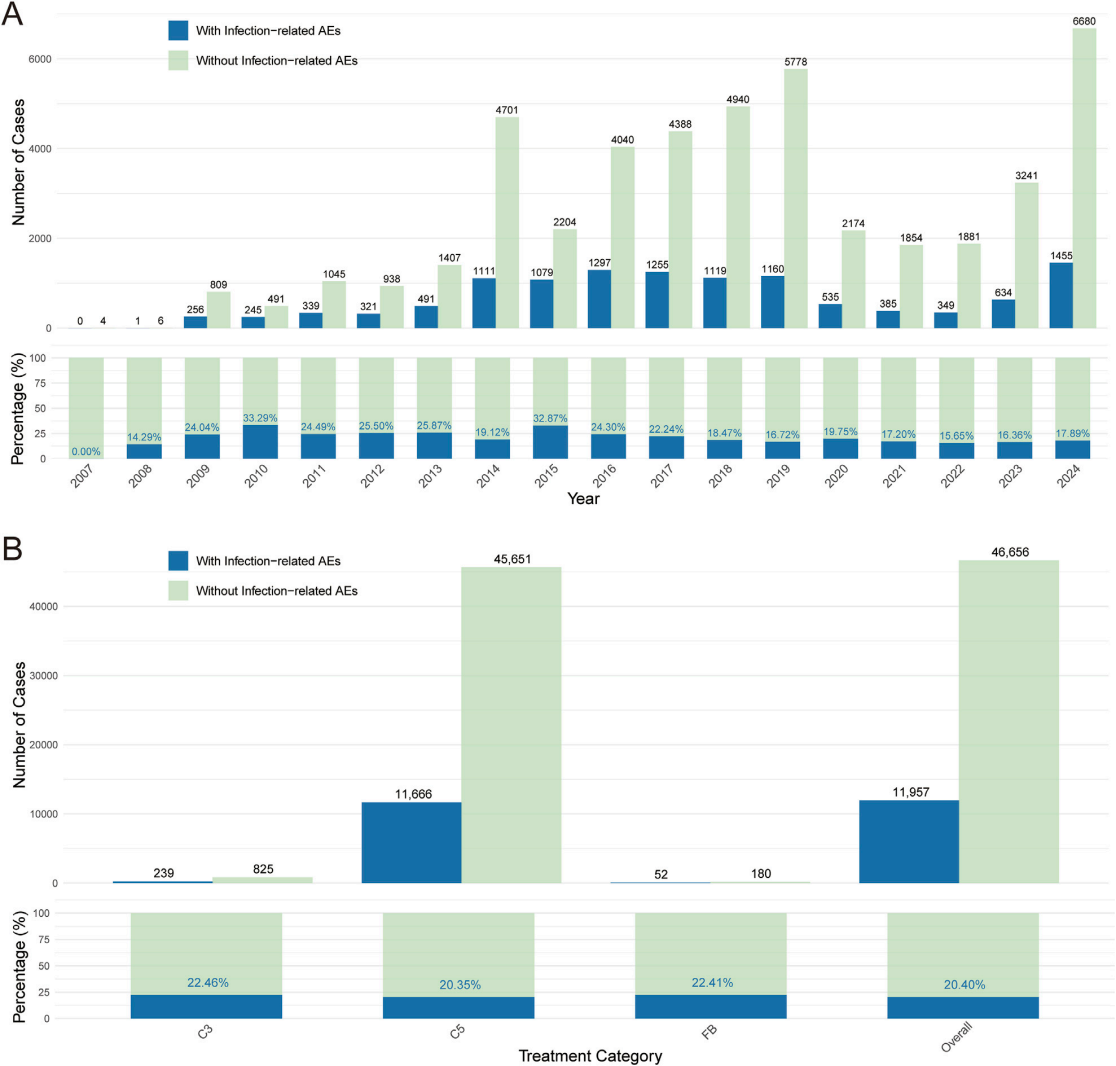
Complement inhibitor-related infectious adverse events. **(A)** Number and proportion of “infection” versus “non-infection” cases among complement inhibitor-related adverse event reports from 2007 to 2024. **(B)** Comparison of the number and proportion of infection and non-infection adverse events under C3 inhibitor, C5 inhibitor, and Factor B inhibitor treatment strategies. Blue represents reports with infection-related adverse events; green represents reports without infection-related adverse events.

To evaluate the potential infectious adverse events associated with use of complement inhibitors, we summarized reports from 2004 to 2024 (no reports from 2004–2006) in FAERS. As shown in Figure 2A, the proportion of infection-related adverse events among complement inhibitor-associated cases increased annually (except the COVID-19 pandemic period from 2020–2022), in line with the expanding clinical application of these agents. We further analyzed the number and proportion of infection-related adverse event reports under different complement inhibitor categories (C3 inhibitors, C5 inhibitors, and Factor B inhibitors) (Figure 2B) and found that a higher number of reports were associated with C5 inhibitor treatment, which might be related to the broad indications and higher clinical usage volume of this type of inhibitor.

### 3.3 Viral infection adverse events associated with complement inhibitors

**FIGURE 3:**
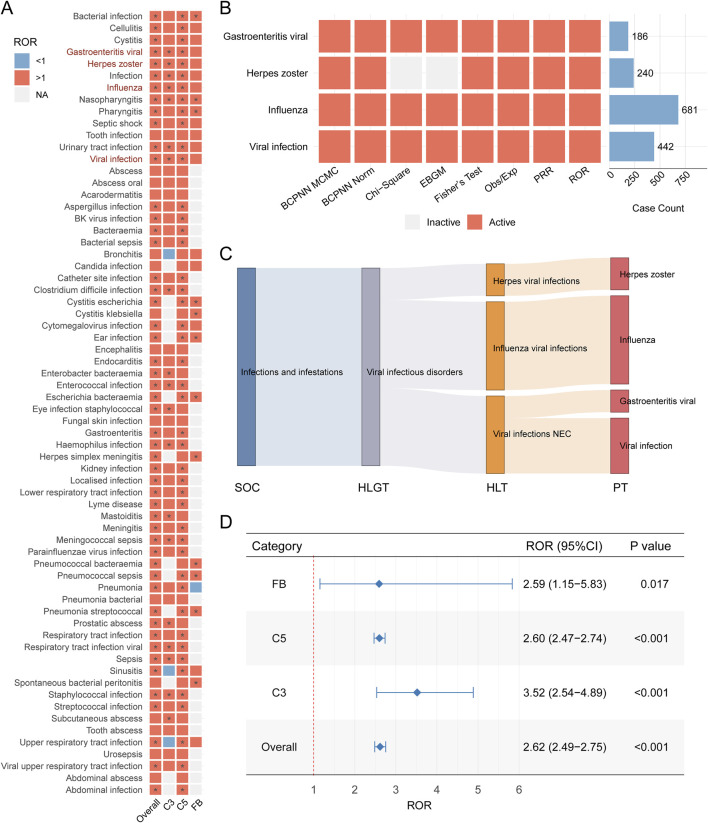
Complement inhibitor-associated viral infection adverse events in FAERS. **(A)** Heatmap of Reporting Odds Ratios (ROR) for viral infection Preferred Terms (PTs). Red indicates ROR>1, blue indicates ROR<1, gray indicates not calculable; * indicates signal detected by ≥ 1 method (full criteria in Methods). **(B)** Complement inhibitor-related viral infection adverse events (CIRVI-AEs) signals across 8 pharmacovigilance metrics. **(C)** Sankey diagram mapping CIRVI-AEs to MedDRA hierarchy: System Organ Class (SOC), High-Level Group Term (HLGT), High-Level Term (HLT), Preferred Term (PT). **(D)** Forest plot comparing category-specific RORs (95% CI) for CIRVI-AEs.

We then focused on viral infection related adverse event among all reported inflection related cases to identify the potential signal indicating viral infection. To achieve this, we conducted pharmacovigilance signal analysis (Figure 3). After applying our criteria for identifying valid signals, we observed that different infection-related adverse events were associated with various complement inhibitor treatment strategies. Ultimately, four preferred terms for viral infections—influenza, herpes zoster, viral gastroenteritis, and viral infection—were identified as being strongly associated with complement inhibitor treatment in the overall analysis (Figure 3A). These were therefore defined as complement inhibitor-related viral infection adverse events (CIRVI-AEs). Similar patterns were observed when we utilized other signal detection metrics (PRR, Fisher’s exact test, Obs/Exp, Chi-square test, two BCPNN methods, and EBGM - 8 methods in total, Figure 3B). The Sankey diagram in Figure 3C, based on the MedDRA hierarchy, illustrates the classification pathway of complement inhibitor-related viral infection adverse events from SOC to PT, visually presenting the distribution across higher levels (HLGT, HLT) for different viral infection PTs, with the largest amount belonging to Influenza viral infection, then followed by viral infection NEC and herpes viral infection. Based on the full FAERS database, we recalculated the ROR for CIRVI-AEs. Overall, complement inhibitor treatments were significantly associated with the occurrence of CIRVI-AEs (ROR = 2.62, 95% CI: 2.49–2.75, *P* < 0.001). However, differences were observed across the categories of complement inhibitors (Figure 3D). C3 inhibitor use was associated with higher odds of reporting viral infections (ROR = 3.52, 95% CI: 2.54–4.89, *P* < 0.001).

### 3.4 Descriptive analysis of viral infection adverse events associated with complement inhibitors

**FIGURE 4:**
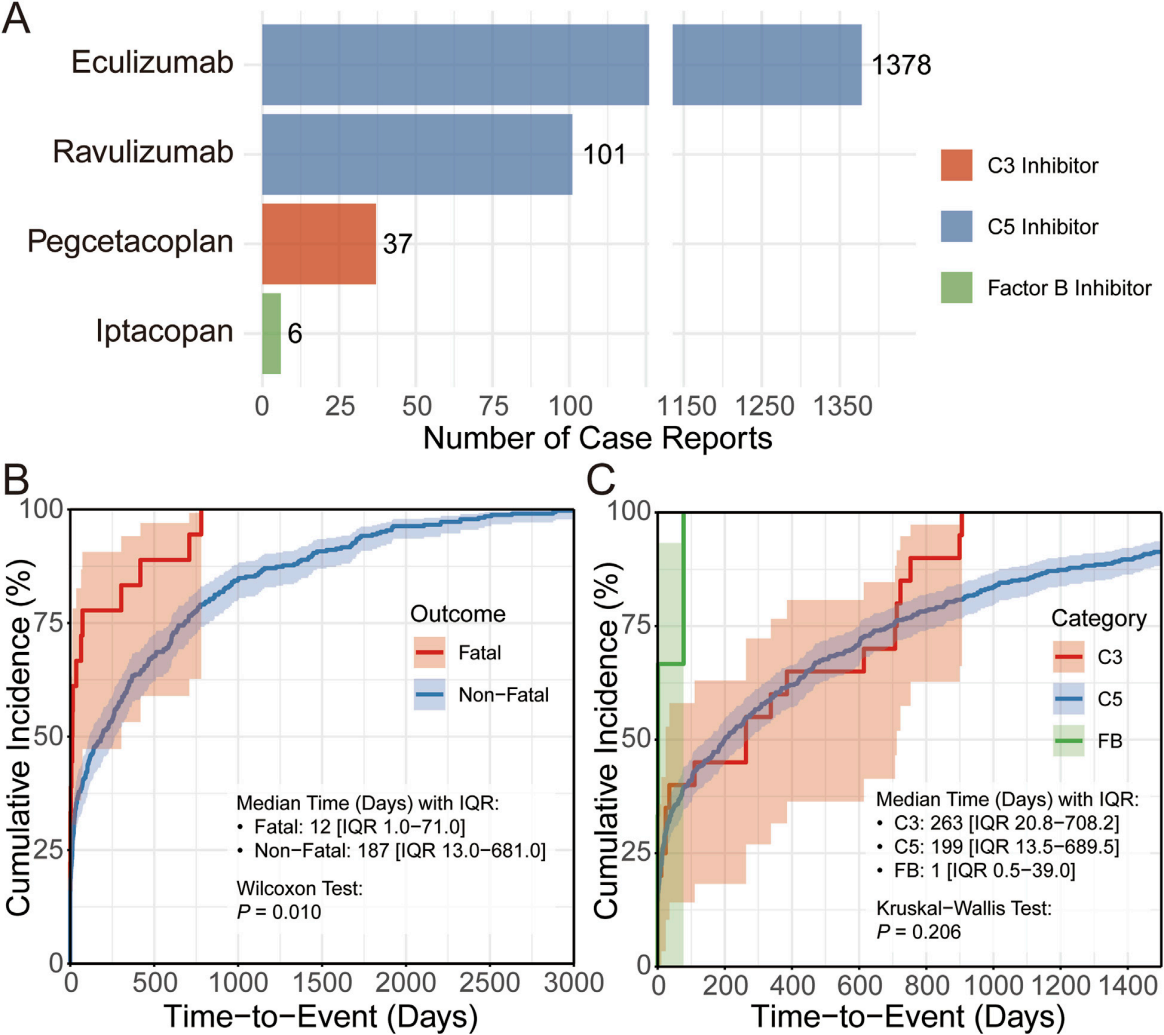
Descriptive analysis of viral infection adverse events associated with complement inhibitors. **(A)** Bar chart showing the number of CIRVI-AEs cases under different complement inhibitor treatment strategies. **(B,C)** Cumulative distribution curves display the time-to-onset distribution of CIRVI-AEs, grouped for comparison by outcome (fatal/non-fatal) and different complement inhibitor treatment strategies.

To further investigate the basic characteristics of complement inhibitor-related viral infection adverse events, we performed detailed descriptive statistical analyses of reported cases across inhibitor category and reaction outcomes. Specifically, we investigated the distribution of case numbers for different complement inhibitors (C3, C5, and Factor B) and the time-to-onset distribution of adverse events in different subgroups (e.g., fatal vs non-fatal cases). As shown in Figure 4A, there exist significant differences in the number of reported viral infection adverse events across cases with different inhibitor groups, with the C5 inhibitor group having markedly more adverse cases than the C3 inhibitor and Factor B inhibitor groups. Further assessment of time-to-onset using cumulative distribution curves revealed distinct temporal patterns between fatal and non-fatal viral infection-related adverse events. Fatal viral AEs occurred much earlier, with a median time-to-event of 12 days, whereas non-fatal viral infections typically manifested later, with a median of 187 days adverse event (Figure 4B). These findings highlight the need for closer patient monitoring and intervention during the early treatment phase, particularly to mitigate severe outcomes. In addition, the median time-to-onset of adverse event differed across three major inhibitor groups, though the overall differences across compared groups were nonsignificant (*P* = 0.206, Figure 4C).

### 3.5 Factors influencing complement inhibitor-associated viral infection adverse events and analysis of fatal outcomes for viral infections compared to other adverse events

**FIGURE 5:**
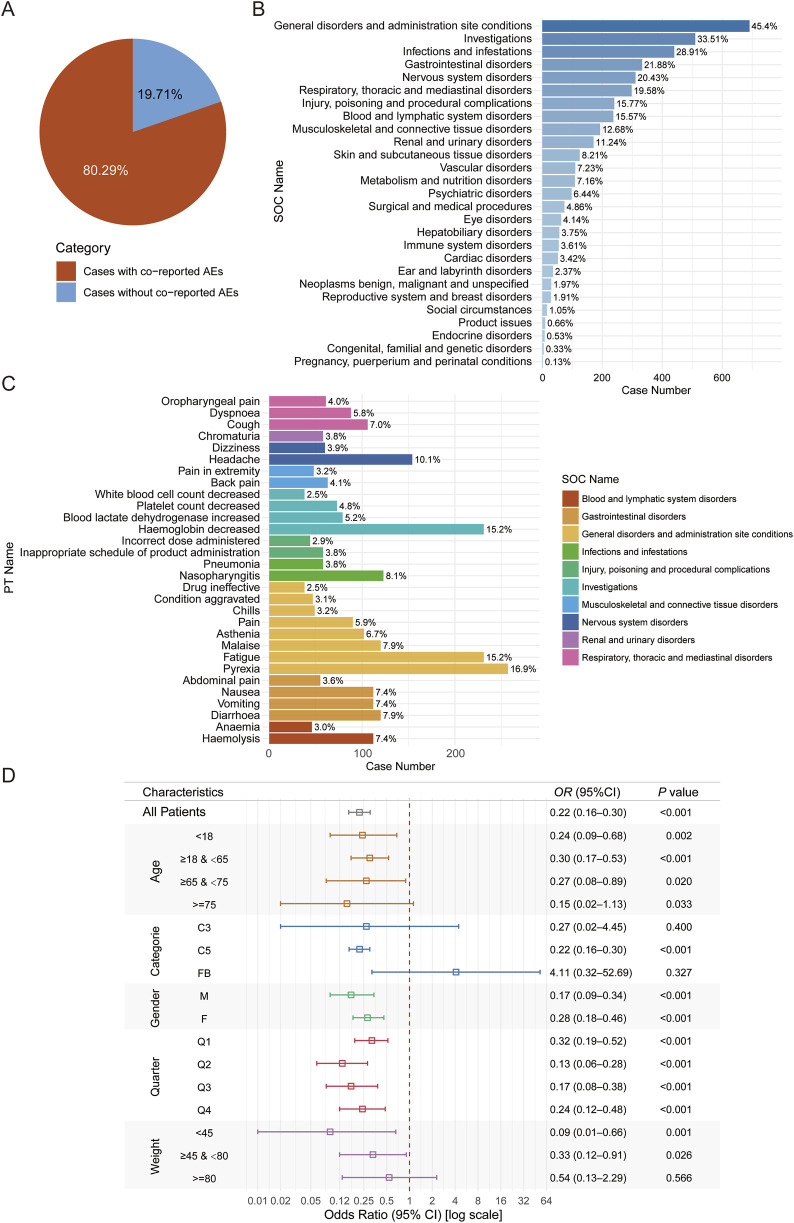
Analysis of factors influencing complement inhibitor-related viral infection adverse events and fatal outcomes for viral infections compared to other adverse events. **(A)** Pie chart showing the proportion of cases with and without concomitant adverse events. **(B)** Bar chart listing the SOC distribution of these concomitant adverse events. **(C)** Displays the 30 most common PTs among concomitant adverse events. **(D)** Forest plot of odds ratios for fatal outcomes in CIRVI-AEs versus non-CIRVI-AEs.

To further explore factors influencing complement inhibitor-associated viral infection adverse events, we analyzed the characteristics of cases with concomitant adverse events. Results indicated that 19.7% of complement inhibitor-related viral infection cases were accompanied by other adverse events (Figure 5A). Further analysis showed that General disorders and administration site conditions (45.4%) is the most common System Organ Class (SOC) for concomitant adverse events, whereas low proportion was observed for pregnancy/perinatal or congenital, familial and genetic disorders (<0.5%) (Figure 5B). Accordingly, among the top 30 most common concomitant adverse event PTs, most of them belong to general disorders and administration site condition, and there are considerable proportions belong to hematology-related PTs (e.g., hemoglobin decreased and haemolysis) (Figure 5C). We further conducted logistic regression to identify potential factors associated with viral infection adverse events. Advanced age exhibited a paradoxical protective effect: patients aged ≥75 years had significantly lower infection risk than those <18 years (OR = 0.43, 95% CI: 0.28–0.66; *P* < 0.001). Female gender was independently associated with increased risk (OR = 1.16, 95% CI: 1.03–1.31; *P* = 0.015). Higher body weight demonstrated a dose-dependent protective effect, with patients weighing 45–80 kg (OR = 0.57, 95% CI: 0.39–0.84; *P* = 0.003) and ≥80 kg (OR = 0.54, 95% CI: 0.35–0.84; *P* = 0.005) showing reduced risk compared to those <45 kg. Seasonal variation was observed, with progressively lower risks in later calendar quarters (Q2: OR = 0.83; Q3: OR = 0.56; Q4: OR = 0.58; all *P* < 0.005 vs. Q1, Supplementary Figure S1). A comprehensive analysis of the FAERS database revealed that complement inhibitor use was associated with reduced mortality across all reported CIRVI-AEs. Patients treated with complement inhibitors exhibited a 4.33% all-cause mortality rate, representing a 32% relative risk reduction (RR = 0.68, 95%CI 0.50–0.93) compared to the 6.34% mortality rate in other therapeutic classes. The robustness of this association was further confirmed by an adjusted odds ratio of 0.67 (95%CI 0.48–0.93). The fatality rate for CIRVI-AEs associated with complement inhibitors was lower compared to other AEs (OR = 0.22, 95% CI: 0.16–0.30; *P* < 0.001, Figure 5D).

### 3.6 Factors influencing fatal outcomes in complement inhibitor-associated infectious adverse events

**FIGURE 6:**
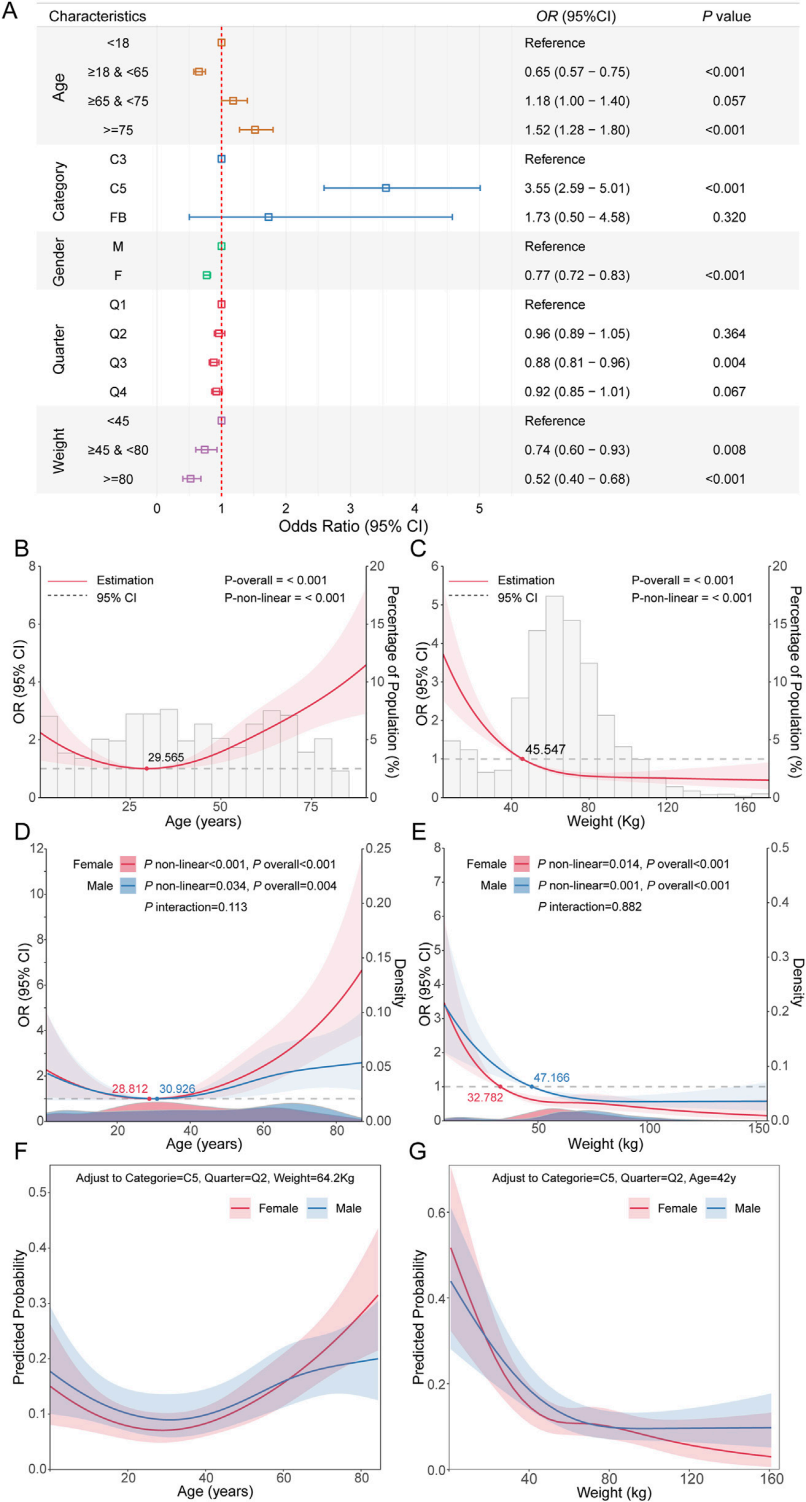
Analysis of factors influencing fatal outcomes in complement inhibitor-related infectious adverse events. **(A)** Forest plot showing odds ratios, 95% confidence intervals, and *P* values from univariate analysis of the impact of major clinical feature on fatal outcomes of infectious adverse events. **(B)** RCS curve showing the relationship between age and the risk of fatal outcomes from infectious adverse events. **(C)** RCS curve for the relationship between weight and the risk of fatal outcomes from infectious adverse events. **(D)** Stratified RCS results showing the relationship between age and fatal outcome risk in different gender subgroups. **(E)** Stratified RCS results showing the relationship between weight and fatal outcome risk in different gender subgroups. **(F)** Probability relationship curves between age and fatal outcomes in different gender subgroups, after adjusting other covariates to reference levels. **(G)** Probability relationship curves between weight and fatal outcomes in different gender subgroups, after adjusting other covariates to reference levels.

To further explore the factors influencing fatal infection-related adverse events associated with complement inhibitors, we performed univariate logistic regression and RCS analyses to evaluate the associations of age, body weight, sex, and complement inhibitor type with the risk of fatal outcomes. Univariate logistic regression revealed significant differences in fatal infection risks among different types of complement inhibitors. Patients treated with C5 inhibitors had a significantly higher risk of fatal adverse events compared to those treated with C3 inhibitors (OR = 3.55, 95% CI: 2.59–5.01, *P* < 0.001; Figure 6A). Female sex (OR = 0.77, 95% CI: 0.72–0.83, *P* < 0.001) and higher body weight were identified as protective factors. Additionally, individuals in the third quarter had a significantly reduced risk of fatal events (OR = 0.88, 95% CI: 0.81–0.96, *P* = 0.004), suggesting a potential protective effect associated with seasonal variation. Subgroup analysis of age also suggested a non-linear relationship with fatal risk. Using RCS modeling, we found that age exhibited a U-shaped association with fatality risk (P-non-linear <0.001), with the lowest risk observed around 29.565 years of age. Risk declined before this point and increased sharply thereafter (Figure 6B). Univariate analysis also supported this observation: individuals aged 18–64 years had a significantly lower risk compared to those <18 years (OR = 0.65, 95% CI: 0.57–0.75, *P* < 0.001), while those ≥75 years showed markedly increased risk (OR = 1.52, 95% CI: 1.28–1.80, *P* < 0.001), indicating age as a bidirectional factor. For body weight, an L-shaped relationship was observed (P-non-linear <0.001), suggesting that low body weight was associated with elevated fatality risk (Figure 6C). We also examined potential interactions between age and sex, as well as body weight and sex. Interaction tests between age and sex, as well as body weight and sex, were not statistically significant (Figures 6D,E), indicating no evidence that the effects of age or weight on fatality risk differed by sex. Consistent trends across sex groups were also observed in stratified analyses, supporting the absence of significant effect modification. Finally, after adjusting for complement inhibitor type, body weight, and season as reference levels, we explored the associations of age and body weight with the probability of infectious fatal adverse event in different sex subgroups (Figures 6F,G).

## 4 Discussion

The therapeutic landscape for complement-mediated diseases (e.g., PNH) has expanded significantly with the development of inhibitors targeting various points in the cascade, including C5, C3, and Factor B (Ricklin et al., 2019; Morgan and Harris, 2015). While these agents confer significant clinical benefits, their interference with complement pathways introduces inherent risks, notably in compromising host immunity against infections (Merle et al., 2015; Ladhani et al., 2019). This study utilized the data from FAERS to perform a comprehensive pharmacovigilance analysis, spanning 2004 to 2024, to delineate the adverse events profile of complement inhibitors, with a particular focus on infectious complications, including viral events. Our investigation distinctively focused on three aspects: providing a broad real-world descriptive overview of AEs, scrutinizing the nuanced profile of viral infections including their associated fatality, and identifying risk factors for overall fatal infectious outcomes. These findings illuminate critical safety signals and contribute novel insights into the real-world implications of complement inhibition. Our descriptive analysis indicated that AE reports predominantly involved adults, with a female preponderance, originating primarily from Americas, and submitted mostly by customers and healthcare professionals. These patterns likely reflect regional prescribing trends, therapeutic access, and heightened reporting vigilance in developed markets. Notably, 8.91% of reported cases resulted in fatality, signaling the potential gravity of AEs in this population. Direct comparisons with clinical trial or registry-derived mortality rates are confounded by methodological disparities (Hughes et al., 2002); nonetheless, this proportion underscores the serious risks accompanying complement inhibition. For instance, while these therapies have mitigated thrombosis-related mortality in paroxysmal nocturnal hemoglobinuria mortality (Kelly et al., 2024), survival outcomes in treated patients may still fall short of matched controls (Hillmen et al., 1995).

Infections emerged as a leading AE category, consistent with the complement system’s pivotal role in innate immunity (Atkinson et al., 2019). The increasing trend in infection reports over time likely parallels the expanding utilization of these agents. But it is important to note that the COVID-19 pandemic caused a temporary decline in the trend from 2020 to 2022, likely due to healthcare service disruptions, reduced patient visits, and impacted reporting systems. C5 inhibitors accounted for the majority of infection-related reports, but this proportion is almost certainly over-estimated because C5 inhibitors are used not only for PNH but also for several other indications. A closer inspection of Figure 2 reveals no evident rise in infection-related adverse events—the outcome under evaluation—suggesting that the higher absolute number of reports reflects the drugs’ extensive market presence rather than an inherently greater risk (Théophile et al., 2011). Notably, a meta-analysis of eculizumab randomized controlled trials (RCTs) reported elevated risks for specific infections (e.g., bacteremia, urinary tract infections) but no significant overall increase relative to controls (Jiang et al., 2025). In contrast, our FAERS analysis, capturing diverse real-world experiences, reinforces infections as a prominent safety concern. Mandatory Risk Evaluation and Mitigation Strategies (REMS) and vaccination protocols for encapsulated bacteria across inhibitor classes further highlight this recognized risk (Ter Avest et al., 2025; White et al., 2025).

A key unique focus of our study was the systematic evaluation of viral risks. Employing robust pharmacovigilance algorithms (Montastruc et al., 2011; Bate and Evans, 2009), we detected significant disproportionality signals for viral infections, including influenza, herpes zoster, and gastroenteritis viral, most notably with C5 inhibitors. While bacterial infections, particularly meningococcal disease, are well-established risks of C5 inhibition (Ladhani et al., 2019), viral infections have garnered less scrutiny (Jiang et al., 2025). The scarcity of systematic FAERS-based evaluations of viral risks across complement inhibitor classes positions our findings as a substantive addition to the literature. However, cross-database or cross-drug class comparisons of signal strength (e.g., Reporting Odds Ratios [ROR]) are limited by data heterogeneity (Hillmen et al., 1995; Montastruc et al., 2011).

Intriguingly, stratified ROR analysis revealed that C3 inhibitors exhibited significantly higher odds of viral infection reports relative to other classes (C5 and Factor B). This disparity suggests distinct reporting profiles or possibly differential biological effects. Given the paucity of long-term comparative data between C3 and C5 inhibitors (White et al., 2025), this observation remains exploratory. Hypotheses from other contexts, such as COVID-19, propose that upstream C3 blockade may exert broader immunological effects than terminal C5 inhibition, potentially influencing viral susceptibility (Mastellos et al., 2020). Further research is imperative to substantiate these preliminary findings. Temporal analysis disclosed that most viral infections manifested within 30 days of treatment initiation, aligning with patterns observed in other targeted therapies (Le et al., 2025; Sun et al., 2025). This early onset suggests a window of heightened vulnerability post-therapy commencement. The shorter median time-to-onset in fatal cases amplifies the clinical relevance of this finding, necessitating confirmation in future studies.

Perhaps the most unexpected observation was the dual protective signature of complement inhibitors in viral infections: not only were CIRVI-AEs less fatal than other complement inhibitor-associated adverse events, but their mortality rate was also significantly lower than same viral infections reported with other drugs. While infections remain a critical safety concern for complement inhibitors, the lower fatality rate for viral infections specifically may indicate a potentially protective effect of complement inhibitors against viral infections, possibly even suggesting an antiviral potential through indirect immunomodulatory mechanisms. This observation from real-world pharmacovigilance data currently lacks direct corroboration in published literature specifically comparing fatality rates across AE types for complement inhibitors. While this lower viral fatality might seem counterintuitive given their immunosuppressive properties, several hypotheses could be considered. Firstly, mortality in many severe viral infections often results from an overexuberant host inflammatory response (i.e., “cytokine storm”) leading to organ damage, rather than direct viral cytopathy; complement inhibitors, by modulating aspects of this inflammatory cascade, might paradoxically mitigate such life-threatening hyper-inflammation (Tisoncik et al., 2012; Vaninov, 2020). This mechanism, where an intense immune reaction dictates severity, has been observed in the mortality patterns of certain patient groups during the COVID-19 pandemic (Risitano et al., 2020; Yang et al., 2021). Secondary, individuals with complement related diseases, such as PNH, may possess an inherently altered baseline immune reactivity that, while contributing to their primary disease, coincidentally renders them less susceptible to the extreme, often fatal, hyper-inflammatory responses observed in other populations during certain viral infections (Bektas et al., 2020; Fleischman, 2015). These proposed mechanisms remain speculative at this stage and underscore the complexity of interpreting severity from spontaneous reports like those in FAERS (Routledge and Bracchi, 2023). The apparent dissociation between infection risk and mortality raises critical questions about clinical management. If complement inhibitors attenuate viral pathogenicity by tempering inflammatory cascades rather than directly enhancing viral clearance, this might imply that primary viral prophylaxis is unnecessary when using complement inhibitors, even in the post-COVID era. However, this hypothesis must be weighed against the elevated infection reporting rates observed with C3 inhibitors. Of note, our finding prompts a crucial question regarding the clinical management of complement inhibitor related AEs, particularly concerning the necessity and scope of routine viral prophylaxis beyond current standards, and merits urgent validation through prospective or registry-based studies (Centers for Disease Control and Prevention (CDC), 2011). Among infection-related AEs, advanced age (>75 years) and C5 inhibitor use emerged as significant predictors of fatal outcomes. The age association reflects broader trends of increased infection severity in older populations, while the C5 inhibitor link may relate to severe bacterial risks (e.g., meningococcal infections) (Ladhani et al., 2019) or differences in patient demographics and exposure duration. Conversely, female gender and higher body weight appeared protective, warranting further exploration.

The strengths of this study include its large scale, leveraging real-world post-marketing data that spans 17 years, and the rigorous application of multiple signal detection methodologies. Nonetheless, the inherent limitations of passive surveillance systems like FAERS are significant (Hughes et al., 2002; Théophile et al., 2011; Goldman, 1998), including reporting biases, data quality issues, inability to calculate true incidence rates, and challenges in establishing causality. Also, prior vaccination status was not specified. Comparisons with findings from other studies, particularly (Jiang et al., 2025) or registries (Sun et al., 2025), must always consider these methodological differences. Meanwhile, this study only includes a population reported as being from the United States , despite the well-known fact that different ethnic groups exhibit distinct complications—such as thrombosis and cytopenias—which may increase the risk of infection, particularly in East Asian populations.

## 5 Conclusion

In conclusion, this FAERS analysis, with its distinct focus on complement inhibitors used reaffirms infections as a central safety concern while offering novel evidence regarding viral risks. Significant signals for specific viral infections signals were most commonly reported with C5 inhibitors but showed the strongest statistical association with C3 inhibitors. The early onset of viral infections and, critically, our unique finding of their lower associated fatality rates compared both to other complement inhibitor-related adverse events and to viral infections reported with other drug, are provocative observations requiring validation but suggest a re-evaluation of their perceived threat in this population. Furthermore, the identification of advanced age and C5 inhibitor use as risk factors for fatal infectious outcomes provides actionable insights for managing patients with completement related diseases, such as PNH. These results advocate for sustained pharmacovigilance, underscore the need for comparative safety research across inhibitor classes, and highlight the role of such focused analyses in generating critical hypotheses for future investigations.

## Data Availability

The original contributions presented in the study are included in the article/Supplementary Material, further inquiries can be directed to the corresponding author.

## Supplementary material

SUPPLEMENTARY FIGURE S1 Forest plot, based on univariate logistic regression results, showing the influence (OR and 95% CI) of different characteristics on the occurrence of viral infection adverse events.
