# Supplementary figures and images for: Viral infections and related fatal adverse events associated with complement inhibitors for PNH: a real-world pharmacovigilance analysis in FAERS
Source: Front Pharmacol. 2025 Aug 11;16:1639685. doi: 10.3389/fphar.2025.1639685 (PMC12375884; doi:10.3389/fphar.2025.1639685)

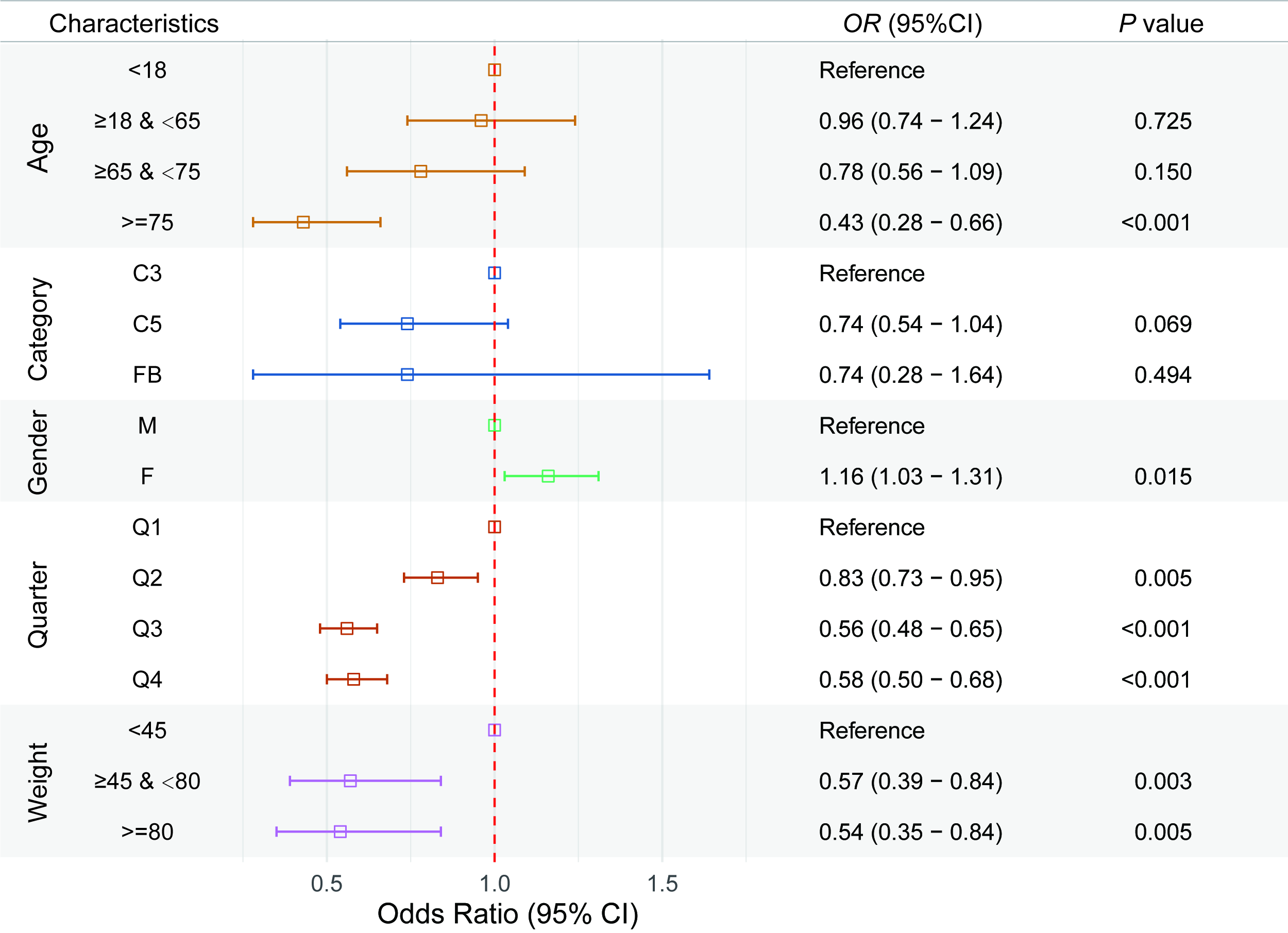

Supplement: Supplementary file 1 [file Image1.tif]
